# Improvement of Acetaldehyde Production in *Zymomonas mobilis* by Engineering of Its Aerobic Metabolism

**DOI:** 10.3389/fmicb.2019.02533

**Published:** 2019-11-14

**Authors:** Uldis Kalnenieks, Elina Balodite, Steffi Strähler, Inese Strazdina, Julia Rex, Agris Pentjuss, Katsuya Fuchino, Per Bruheim, Reinis Rutkis, Katherine M. Pappas, Robert K. Poole, Oliver Sawodny, Katja Bettenbrock

**Affiliations:** ^1^Institute of Microbiology and Biotechnology, University of Latvia, Riga, Latvia; ^2^Max-Planck-Institute for Dynamics of Complex Technical Systems, Analysis and Redesign of Biological Networks, Magdeburg, Germany; ^3^Institute for System Dynamics, University of Stuttgart, Stuttgart, Germany; ^4^Department of Biotechnology and Food Science, NTNU Norwegian University of Science and Technology, Trondheim, Norway; ^5^Department of Genetics and Biotechnology, Faculty of Biology, National and Kapodistrian University of Athens (NKUA), Athens, Greece; ^6^Department of Molecular Biology and Biotechnology, The Krebs Institute, University of Sheffield, Sheffield, United Kingdom

**Keywords:** *Zymomonas mobilis*, acetaldehyde, metabolic engineering, stoichiometric model, metabolomics

## Abstract

Acetaldehyde is a valuable product of microbial biosynthesis, which can be used by the chemical industry as the entry point for production of various commodity chemicals. In ethanologenic microorganisms, like yeast or the bacterium *Zymomonas mobilis*, this compound is the immediate metabolic precursor of ethanol. In aerobic cultures of *Z. mobilis*, it accumulates as a volatile, inhibitory byproduct, due to the withdrawal of reducing equivalents from the alcohol dehydrogenase reaction by respiration. The active respiratory chain of *Z. mobilis* with its low energy-coupling efficiency is well-suited for regeneration of NAD^+^ under conditions when acetaldehyde, but not ethanol, is the desired catabolic product. In the present work, we sought to improve the capacity *Z. mobilis* to synthesize acetaldehyde, based on predictions of a stoichiometric model of its central metabolism developed herein. According to the model analysis, the main objectives in the course of engineering acetaldehyde producer strains were determined to be: (i) reducing ethanol synthesis via reducing the activity of alcohol dehydrogenase (ADH), and (ii) enhancing the respiratory capacity, either by overexpression of the respiratory NADH dehydrogenase (NDH), or by mutation of other components of respiratory metabolism. Several mutants with elevated respiration rate, decreased alcohol dehydrogenase activity, or a combination of both, were obtained. They were extensively characterized by determining their growth rates, product yields, oxygen consumption rates, ADH, and NDH activities, transcription levels of key catabolic genes, as well as concentrations of central metabolites under aerobic culture conditions. Two mutant strains were selected, with acetaldehyde yield close to 70% of the theoretical maximum value, almost twice the previously published yield for *Z. mobilis*. These strains can serve as a basis for further development of industrial acetaldehyde producers.

## Introduction

Acetaldehyde is a valuable product of microbial biosynthesis, which can be used by chemical industry as the entry point for the synthesis of acetic anhydride, acetic acid, butadiene, butanol, crotonaldehyde, and other commodity chemicals (Danner and Braun, 1999; Moore et al., 2017). Also, acetaldehyde is an important aroma compound in yogurt and other dairy products (Bongers et al., 2005). In microorganisms, this compound is the metabolic precursor of ethanol. In aerobic cultures of ethanologenic microorganisms, it accumulates as a volatile, inhibitory byproduct because of withdrawal of reducing equivalents from the alcohol dehydrogenase reaction by respiration. Partly due to its toxicity, aerobic cultures tend to convert acetaldehyde into less toxic products: reduce it to ethanol or oxidize it to acetate depending on the culture conditions. Hence, <50% of the theoretical yield has been reached in acetaldehyde bioprocesses so far (Wecker and Zall, 1987; Bongers et al., 2005; Balagurunathan et al., 2018), leaving a considerable space for yield improvement.

Microorganisms, like *Zymomonas mobilis*, genetically engineered *Lactococcus lactis* and *Escherichia coli* have been studied for production of acetaldehyde from sugary substrates (Wecker and Zall, 1987; Bongers et al., 2005; Balagurunathan et al., 2018). The advantage of *Z. mobilis* as an acetaldehyde producer lies in the high rate of its Entner-Doudoroff (ED) glycolytic pathway, the simplicity of its central metabolism with an incomplete pentose phosphate pathway (PPP) and a truncated tricarboxylic acid cycle (TCA), which serve only for biosynthetic purposes, and its very active pyruvate decarboxylase. Besides, *Z. mobilis* exhibits low biomass production with only 2–5% of the consumed substrate carbon being converted into precursors for biomass synthesis, thus providing a solid basis for high product yields (Swings and De Ley, 1977; Rogers et al., 1982; De Graaf et al., 1999). Furthermore, for some reason, *Z. mobilis* does not use its respiration to supply energy for aerobic growth in the same way as the majority of aerobic and facultative anaerobic microorganisms do (Belauïch and Senez, 1965; Rutkis et al., 2014), which makes it unique among the producer microorganisms. The active respiratory chain with its low energy-coupling efficiency is ideally suited for regeneration of NAD^+^ under condition when acetaldehyde, but not ethanol, is the major catabolic product (Kalnenieks, 2006; Rogers et al., 2007).

Several stoichiometric models of *Z. mobilis* metabolism based on genomic annotations have been published so far (Tsantili et al., 2007; Lee et al., 2010; Widiastuti et al., 2011; Motamedian et al., 2016). The medium-scale model from Pentjuss et al. (2013), which focuses on *Z. mobilis* central metabolism, entails certain advantages: it is based on the aforementioned genome-scale reconstructions but was supplemented by further biochemical evidence concerning special features of this bacterium regarding the NAD(P)H balance and its aerobic metabolism. Therefore, we chose this model for our metabolic engineering approach with the aim to improve the *Z. mobilis* acetaldehyde yield. For this purpose, considering only the central metabolism is sufficient since it represents the primary target for genetic engineering approaches.

In the present work, we improved the ability of *Z. mobilis* to synthesize acetaldehyde, based on interventions predicted by the stoichiometric model that was complemented by reactions describing biomass synthesis. The aerobic metabolism was identified as the primary target and the main objectives for genetic engineering were determined to be a reduction of the fermentative metabolism, mainly ethanol synthesis via alcohol dehydrogenase (ADH), in favor of respiration that was further enhanced by overexpression of the respiratory NADH dehydrogenase (NDH). Several mutants were extensively characterized by determining growth rates, product yields, oxygen consumption, respiratory rates, ADH as well as NDH activity, expression levels of key enzymes and by metabolomics.

## Materials and Methods

### Strains Used in the Study

Plasmids and strains used in the present work are listed in Table 1. *Escherichia coli* JM109 was purchased from Promega (Madison, USA). Strain JM109 was used as the host for cloning of the recombinant plasmids.

**Table 1:** Plasmids and strains used in this study.

**Plasmid/strain**	**Characteristics**	**Source**
pBT	Plasmid vector, Cm^r^	Stratagene
pGEM-3Zf(+)	Plasmid vector, Amp^r^	Promega
pBBR1MCS-2	Plasmid vector, Kan^r^	Addgene
pNDH	pBBR1MCS-2 derivative, carrying an 1.5 kb fragment of PCR-amplified type II NADH dehydrogenase gene *ndh* (ZZ6_RS01100) with its promoter region, cloned between the BamHI and HindIII sites of the plasmid MCS	Rutkis et al., 2016
pGEM*cat*::cm^r^	pGEM-3Zf(+) derivative, carrying an 0.8 kb PCR-amplified fragment of pBT with 0.7 kb of the chloramphenicol resistance gene, inserted into the *Eco*RI site of the 1.4 kb fragment of the *Z. mobilis* catalase gene *cat* (ZZ6_RS01975), cloned between the *Hind*III and *Bam*HI sites of the plasmid MCS	Strazdina et al., 2012
Zm6	Parent strain	ATCC 29191
Zm6-*cat*	Zm6 strain with a Cm^r^ insert in the ORF of *cat*	Strazdina et al., 2012
Zm6-*adhB*	Zm6 strain with a Kan^r^ insert in the ORF of the iron-containing alcohol dehydrogenase gene *adhB* (ZZ6_RS07070)	Kalnenieks et al., 2006
Zm6-*cat* /pNDH	*Zm6-cat*, transformed with the pNDH plasmid	Present work
Zm6/pNDH	Zm6 strain transformed with the pNDH plasmid	Rutkis et al., 2016
Zm6-*adhB*-*cat*	Zm6 strain with a Kan^r^ insert in the ORF of *adhB* and Cm^r^ insert in the ORF of *cat*	Present work
Zm6-*ndh*	Zm6 strain with a Cm^r^ insert in the ORF of *ndh*	Kalnenieks et al., 2008

*Zymomonas mobilis* was transformed by electroporation (Liang and Lee, 1998). Genomic DNA isolation from *Z. mobilis*, PCR and cloning techniques were described before (Kalnenieks et al., 2006, 2008). Strain Zm6-*cat* was transformed with the pNDH plasmid, yielding strain Zm6-*cat*/pNDH. That was done following the same routines as previously described for overexpression of NDH in Zm6 (Rutkis et al., 2016). The double knock-out strain Zm6-*adhB*-*cat* was obtained by transforming the strain Zm6-*adhB* with the pGEMcat::cm^r^ plasmid and selection of homologous recombinants by the same procedures as described for construction of the *cat* single knock-out strain (Strazdina et al., 2012).

*Zymomonas mobilis* strains were maintained and cultivated without shaking at 30 °C in a growth medium (ZM medium) containing glucose (50 g/l), yeast extract (5 g/l), and mineral salts, as described previously (Kalnenieks et al., 1993), with addition of the relevant selective antibiotics (Kalnenieks et al., 2006; Strazdina et al., 2012; Rutkis et al., 2016). For screening of enzymatic activities in the strains, aerated batch cultivations were carried out on a shaker (New Brunswick Scientific Incubator shaker, series I 26) at 155 rpm, in 500 ml unbaffled flasks containing 50 ml culture without antibiotic addition. Cell concentration was determined spectrophotometrically as OD_600_ and dry cell mass of the suspensions was calculated by reference to a calibration curve. For this 10 ml of culture was centrifuged for 20 min at 5,000 rpm, the cell pellet was washed with water and afterwards dried at 105°C for 24 h. The mass of the cell pellet was determined by the weight difference between the dried tube with and without cells. Using this method 1 OD_600_ corresponded to 0.24 g_cdw_ × l^−1^.

### Enzymatic Assays and Monitoring of Respiratory Activity

Whole cell oxygen uptake was measured for samples taken directly from shaken flasks and transferred into the chamber of a Clark-type oxygen electrode (Kalnenieks et al., 2000). For preparation of cell-free extracts, cells were sedimented by centrifugation at 5,000 rpm for 15 min, resuspended in 100 mM potassium phosphate buffer containing 2 mM magnesium sulfate, pH 6.9, and disrupted by disintegration with abrasive quartz beads, 125–150 mm diameter, in a homogenizer at 30 Hz for 2.5 min. Separation of cytoplasmic membranes was performed by ultracentrifugation as described previously (Kalnenieks et al., 1993). Protein concentration in cell-free extracts and membrane samples was determined according to Markwell et al. (1978). The NADH oxidase assay for membranes was carried out by following NADH oxidation spectrophotometrically at 340 nm, as previously described (Kalnenieks et al., 2008). NADH dehydrogenase activity of membranes was measured after the terminal oxidase was inhibited by addition of 20 mM KCN and using ubiquinone Q_1_ as the electron acceptor (Strazdina et al., 2018). Alcohol dehydrogenase isoenzyme activities were monitored as described in Kalnenieks et al. (2006).

### Cultivation

For growth assays colonies of the respective *Z. mobilis* strain were inoculated in ZM medium and incubated at 30°C in closed tubes overnight. This pre-culture was washed and used to inoculate either another tube containing 40 ml fresh ZM medium or a bioreactor containing 400 ml fresh ZM medium to an OD_600_ of about 0.2–0.3. For anaerobic growth assays, closed tubes were incubated at 30°C without agitation. For aerobic bioreactor experiments cells were incubated at 30°C under strong aeration (1 vvm air) and stirred at a rate of 500 rpm. Bioreactors were equipped with pO_2_ and pH probes as well as with online off-gas analysis (BlueSens, Herten, Germany) allowing for the analysis of dissolved oxygen, as well as CO_2_ production and O_2_ uptake. At regular intervals, OD_600_ was determined and samples for analysis of supernatants and off-gas were taken.

### Product Assays

Samples from growth curves were quickly centrifuged and the supernatant was analyzed with respect to glucose content using the D-glucose HK assay kit (Megazyme International, Ireland), ethanol using an ethanol assay kit (Megazyme International, Bray, Ireland) and acetaldehyde using the acetaldehyde assay kit (Megazyme International, Bray, Ireland) according to the manufacturer's information. Assays were performed in 96-well plates.

For analysis of acetaldehyde and acetoin samples were derivatized with 2,4-dinitrophenylhydrazin similar to Pezzoli et al. (1984). Basically, 590 μl of sample were mixed with 410 μl derivatization mix (24 mM H_2_PO_4_, 1.48 mg/ml 2,4-dinitrophenylhydrazin in acetonitrile). Samples were incubated for at least 5 min at room temperature and subjected to HPLC analysis (Agilent 1100 Series LC/MSD; Detector 1946/1956 MSD, Agilent, Santa Clara, CA, USA) using a Lichrosorb 10 μm RP-18 100A column (Phenomenex, Aschaffenburg, Germany) eluted with 50% acetonitrile at a flow rate of 1 ml/min at 40°C. Samples were quantified based on absorption at 365 nm.

For analysis of acetaldehyde in the off-gas, the off-gas was led through a glass vial filled with 5 ml derivatization mix for 5–10 s. Afterwards the mix was incubated at room temperature for at least 5 min and afterwards filtered and analyzed by HPLC as described above. By considering the time of sampling, the gas flow and the liquid volume of the culture the amount of acetaldehyde in the off-gas was counted back to the initial concentration in the culture.

For the determination of product yields the amounts of glucose present at different time points were plotted against the corresponding concentrations of the respective product. Yields were determined from the resulting linear correlation.

### Gene Expression Analysis

Quantification of transcript levels based on qRT-PCR was performed as described previously (Nitzschke and Bettenbrock, 2018). About 1.5 × 10^9^ cells from exponential growth phase were quenched in twice the volume of RNAprotect Bacterial Reagent (Qiagen, Hilden, Germany), vortexed for 5 s and incubated at room temperature for 5 min. Cells were pelleted by centrifugation, the supernatant was discarded and the pellet was stored at −80°C. RNA was prepared using the Master Pure RNA Purification Kit (Epicenter, Madison, USA). RNA concentration and purity was determined using the NanoDrop spectrophotometer (Thermo Fisher Scientific).

mRNA was transcribed into cDNA by using the RevertAid H Minus First Strand cDNA synthesis Kit (Thermo Fisher Scientific). Quantitative PCR of different cDNA samples was performed using the MesaGreen qPCR Master Mix Plus (Eurogenetec, Köln, Germany) with SYBR Green as detection agent and the Rotor-Gene 6000 (Qiagen, Hilden, Germany). Amplification conditions were: 95 °C for 10 min, 40 cycles at 95°C for 15 s and 60°C for 1 min. A negative control without template was conducted for each primer pair in each PCR run and a control for DNA contamination was performed for each RNA sample used. Quantification was performed by relative quantification to a housekeeping gene (*gap*) applying the ΔΔCt method (Livak and Schmittgen, 2001; Hellemans et al., 2007) using the R package ddCt (Zhang et al., 2019).

We chose a number of genes for RT-qPCR analysis. The respective genes and primer sequences are shown in Table 2. Gene sequences were chosen from NCBI Reference Sequence NC_006526.2 for ZM4 based on their annotation (Yang et al., 2009) or based on the information from a genome scale model (Motamedian et al., 2016). Sequence identifiers are indicated in Table 2. We note that for pyruvate decarboxylase, respiratory type II NADH dehydrogenase, and for both alcohol dehydrogenase isoenzymes their current genome annotations need clarification. In the NCBI Reference Sequence NC_006526.2 the gene ZMO_RS06080 is annotated as encoding alpha-keto acid decarboxylase family protein, ZMO_RS04970 as encoding NAD(P)/FAD-dependent oxidoreductase, ZMO_RS05560 as encoding alcohol dehydrogenase AdhP, while ZMO_RS07165 as encoding L-threonine dehydrogenase. Based on previous publications on the biochemical characteristics of these enzymes and the according nucleotide sequences of the genes encoding them, we identified ZMO_RS06080 as the pyruvate decarboxylase gene, *pdc* (Conway et al., 1987a), ZMO_RS04970 as the respiratory type II NADH dehydrogenase gene, *ndh* (Kalnenieks et al., 2008), ZMO_RS05560 as the ADH I gene, *adhA* (Keshav et al., 1990), and ZMO_RS07165 as the ADH II gene, *adhB* (Conway et al., 1987b).

**Table 2:** List of genes and primer sequences for gene expression analysis.

**Gene**	**Enzyme**	**PRIMER sequences (5^**′**^…3^**′**^)**
*adhA* (ZMO_RS05560)	Alcohol dehydrogenase I (Zn-containing)	TTGGCAAAAGCGAGCTGTTCATC TGGGCGGTTTAGGCAATCT
*adhB* (ZMO_RS07165)	Alcohol dehydrogenase II (Fe-containing)	AGCCGCGTTTGCCTTATG GCCGGTGTCTTGATCTTGTCTA
*ndh* (ZMO_RS04970)	Type II respiratory NADH dehydrogenase	CAGATGGCCGATACAGTTTTTGA AATGCAGGCGATAGAGCGAGTTA
*ldh* (ZMO_RS01110)	FAD-dependent respiratory lactate dehydrogenase	TTGCGGCGAAATACGGAAAAGAT ATAACGCGGCAAGAAACTGACACG
*ldh* (ZMO_RS05565)	NAD-dependent lactate dehydrogenase	ACTTCGGCATTGGCTTCGTC CGCGTCGCAATATTCAGTTCC
*ald* (Zmob_1390)	Aldehyde dehydrogenase	CGTATCGCGGGACCGTATCTGA GCGCCCGCTTCTTCCAAAAC
*cat* (ZMO_RS04105)	Catalase	TCGCCCTTTGAAAACGCATCCTA AACGGGCTTTTTCATCCTCTTTCA
*gap* (ZMO_RS00775)	Glyceraldehyde 3 P dehydrogenase	ACCCCGGATGTCAGCCTTGTTG GGAGCTGTGCGGATCGGAGTAGAA
*glk* (ZMO_RS01580)	Glucokinase	GCCGCTTTGGATCGCTTCTGCT ACCGACACCACCGCCAATAACAAC
*zwf* (ZMO_RS01570)	Glucose 6 P dehydrogenase	AAGTTGCCGGTTACATTGACGA TGCGGATATAGAACGGAACACC
*cyt* (ZMO_RS07025)	CydA, subunit I of *bd* cytochrome quinol oxidase	CCTTCCCCGATCCTGTTTTTCTCT AGCCCGACGGTGCCATCAATA
*pdc* (ZMO_RS06080)	Pyruvate decarboxylase	AGCTTCTTCCGGGGTGTAAATC GTGGCGCCTATGCAGAAAACCT
*pdh* (ZMO_RS07205)	Pyruvate dehydrogenase, subunit E1	CATTGGGCCCACGGAAGACGAT GTGGGTGCCGCAATGGAAGGTT

### Measurement of Intracellular Metabolites

Cells from aerobic bioreactor experiments were sampled in exponential phase at an OD_600_ of ~1. About 5 ml cells were quickly filtered through Supor PES 0.8 μm filters (Pall, Port Washington, USA) and immediately washed with 10 mL cold saline (1 g/l KH_2_PO_4_, 0.5 g/L MgSO_4_). After an additional wash with 10 ml cold H_2_O the filters were quickly removed and immersed into 13 ml cold 55% acetonitrile. Cells were disrupted by three freeze-thaw cycles in liquid nitrogen and room temperature. Filters were removed and cell debris was pelleted by centrifugation at 0°C. Samples were divided in 4 ml aliquots, frozen in liquid nitrogen and afterwards lyophilized.

Freeze-dried samples were re-suspended in 300 μL LC-MS gradient H_2_O (Waters, Millford, MA, US), spin-filtered through polyethersulfone membrane (VWR, Radnor, PA, USA) by centrifugation (5804R, Eppendorf AG, Hamburg) at 14,000 rpm for 15 min. The filtered samples were analyzed by capillary ion chromatography coupled to tandem mass spectroscopy as previously described (Kvitvang et al., 2014; Stafsnes et al., 2018), except that Triple Quadrupole Mass Spectrometry TQXS (Waters, Millford, MA, USA) was used for detection. The measurement was repeated with two biological replicates and 5–9 technical replicates for each strain. The measurements were corrected by ^13^C labeled internal standards (Stafsnes et al., 2018). The obtained metabolome data were first analyzed by Principal component analysis, using platform offered in MetaboAnalyst (Xia et al., 2009) for simplified visualization of metabolite abundance pattern in each strains. The data were auto-scaled prior to PCA. Obtained scores were plotted in 2-dimensional principal components.

### Stoichiometric Modeling

The constraint-based model is based on the stoichiometric model of *Z. mobilis* central metabolism published by Pentjuss (Pentjuss et al., 2013) and was complemented by reactions describing biomass synthesis according to the published ^13^C-NMR flux analysis (De Graaf et al., 1999). This ensures that the necessary metabolites required for cell growth are considered when analyzing the stoichiometric model for optimal acetaldehyde production. In order to investigate the stoichiometric model by means of flux balance analysis, calculation of a steady state is required. This was not possible when including the reaction for biomass synthesis according to the composition (De Graaf et al., 1999), since an accumulation of erythrose-4-phopshate (E4P) occurred. This can be explained by the fact that the model presented here only depicts the central carbon metabolism and *Z. mobilis* could use E4P for the synthesis of secondary metabolites which is not represented by the model. Therefore, an artificial export reaction (EX_e4p) was introduced that removes any additional E4P from the system. A detailed model description including all reactions and metabolites along with their abbreviations can be found in the supplementary material in Data Sheet 1. The newly added reactions are highlighted in blue.

Flux balance analysis (FBA) was carried out using MATLAB R2018a and the COBRA Toolbox v2.0 (Schellenberger et al., 2011). For all FBAs, the glucose consumption rate as well as biomass export was set constant, while the model was optimized for maximum acetaldehyde yield.

## Results

### Model-Based Optimization of Acetaldehyde Production by *Z. mobilis*

**Figure 1:**
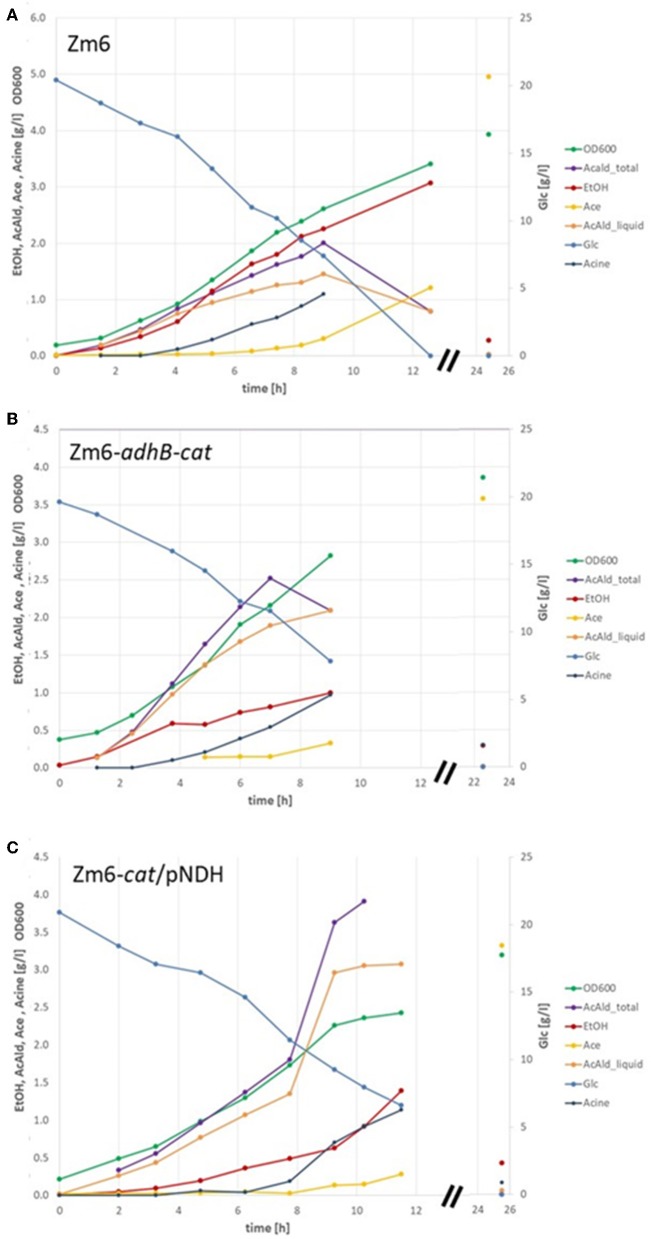
Batch fermentation of WT Zm6 and of acetaldehyde producer strains Zm6-*adhB*-*cat* and Zm6-*cat*/pNDH. Biomass was estimated by measuring the optical density at 600 nm (OD600). The concentrations of glucose (Glc) as substrate and the product acetaldehyde in the liquid (AcAld_liquid) and in total (AcAld_total) as well as the byproducts ethanol (EtOH), acetate (Ace), and acetoin (Acin) were determined by enzymatic assays. **(A)** Shows one selected batch with Zm6, **(B)** with Zm6-*adhB*-*cat*, and **(C)** with Zm6-*cat*/pNDH.

Contrary to when grown in anaerobic batch on glucose and producing only ethanol, the *Z. mobilis* wildtype (WT) strain Zm6 typically produces acetaldehyde and minor amounts of additional products during aerobic conditions. The main product is still ethanol, but also a significant and almost equal production of acetaldehyde is observed (Figure 1A). Acetate production is delayed, probably initiated first after reaching an intracellular threshold level of acetaldehyde, and acetoin is detected only at trace levels. Under aerobic conditions, Zm6 is also able to take up the excreted ethanol and oxidize it to acetic acid after glucose depletion (Figure 1A). These results under aerobic conditions show that *Z. mobilis* can utilize a respiratory chain for regeneration of NAD and does not only rely on a strict fermentative NAD/ NADH balance.

In order to improve the acetaldehyde yield and identify targets for genetic interventions, the flux distribution within the central metabolism of WT Zm6 was analyzed using the MATLAB-based Toolbox Cobra and the previously published stoichiometric model from Pentjuss et al. (2013) with additional reactions describing biomass synthesis (see Materials and Methods). First, a flux distribution was calculated that resembles that of the WT strain under aerobic conditions as measured during batch cultivation on glucose. For that purpose, the export fluxes of biomass, glucose, ethanol, acetate, and acetoin were fixed according to the measured values and the flux distribution for optimal acetaldehyde production was calculated (Table 3, A). In accordance with current knowledge regarding *Z. mobilis* metabolism, most of the consumed carbon is converted to pyruvate via the fast and efficiently functioning ED pathway. Only a small amount, ~5%, is converted into precursors for biomass synthesis via the PPP as well as the TCA routes (Swings and De Ley, 1977; Rogers et al., 1982). As a facultative anaerobic bacterium, *Z. mobilis* does not necessarily require oxygen, however, it exhibits active respiration during aerobic batch cultivation for regeneration of NAD(P)^+^. Nevertheless, for the observed experimental conditions most NAD^+^ is regenerated via the fermentative metabolism by conversion of pyruvate to ethanol via the intermediate product acetaldehyde. Besides, acetaldehyde is also converted into minor amounts of acetate toward the end of the batch process and small portions of pyruvate are converted into acetoin. As a comparison, the flux distribution for the theoretically optimal acetaldehyde production was calculated in WT Zm6. The calculated optimal acetaldehyde yield is 1.86 mol acetaldehyde per mol glucose consumed which occurs if no byproducts are produced (see Table 3, B). In this scenario, glucose is converted into equimolar amounts of acetaldehyde and carbon dioxide minus the carbon that is used for synthesis of biomass precursors. Since the main product of *Z. mobilis* is ethanol, a major improvement of the acetaldehyde yield is anticipated by minimizing the flux through the alcohol dehydrogenase (ADH). The model-based analysis demonstrates that successively reducing the flux through ADH improves acetaldehyde production (Table 3, C), whereby knockout of ADH obviously results in the best acetaldehyde yield close to the theoretical optimum (Table 3, D). The respiration as well as oxygen consumption concomitantly increases with decreased ADH activity. The model-based analysis demonstrates that a similar flux distribution as in the ADH deletion strain can be alternatively obtained by forcing increased oxygen uptake and, thereby, a higher flux through the respiratory system (Table 3, E). In this way, increased amounts of NAD^+^ are regenerated via respiration provoking a reduction in ethanol synthesis in favor of acetaldehyde production.

**Table 3:** Flux distribution of *Z. mobilis* central metabolism for optimal acetaldehyde production during batch fermentation on glucose.

**Reaction**	**Pathway**	**A**	**B**	**C**			**D**	**E**
GLK	ED pathway	1.00	1.00	1.00	1.00	1.00	1.00	1.00
PGI		0.01	0.01	0.01	0.01	0.01	0.01	0.01
G6PDH_nadp		0.99	0.99	0.99	0.99	0.99	0.99	0.99
PGL		0.99	0.99	0.99	0.99	0.99	0.99	0.99
EDD		0.99	0.99	0.99	0.99	0.99	0.99	0.99
KDPGA		0.99	0.99	0.99	0.99	0.99	0.99	0.99
GAPDH		0.98	0.98	0.98	0.98	0.98	0.98	0.98
PGK		0.98	0.98	0.98	0.98	0.98	0.98	0.98
PGM		0.96	0.96	0.96	0.96	0.96	0.96	0.96
ENO		0.96	0.96	0.96	0.96	0.96	0.96	0.96
PYK		0.94	0.94	0.94	0.94	0.94	0.94	0.94
PDC	Fermentation	1.80	1.86	1.80	1.80	1.80	1.80	1.80
ADH		0.68	0.00	**0.60**	**0.40**	**0.20**	**0.00**	0.00
ACDH_nad		0.07	0.00	0.07	0.07	0.07	0.07	0.07
RNDH_nadh	Respiration	0.40	1.01	0.48	0.68	0.88	1.08	1.08
RNDH_nadph		0.77	0.77	0.77	0.77	0.77	0.77	0.77
UO		0.58	0.89	0.62	0.72	0.82	0.92	0.92
TKT2	PPP	−0.01	−0.01	−0.01	−0.01	−0.01	−0.01	−0.01
RPE		0.01	0.01	0.01	0.01	0.01	0.01	0.01
RPI		0.01	0.01	0.01	0.01	0.01	0.01	0.01
PDH	TCA	0.03	0.03	0.03	0.03	0.03	0.03	0.03
EX_glc	Export	**−1.00**	**−1.00**	**−1.00**	**−1.00**	**−1.00**	**−1.00**	**−1.00**
EX_o2		−0.58	−0.89	−0.62	−0.72	−0.82	−0.92	**−0.92**
EX_biomass		**0.01**	**0.01**	**0.01**	**0.01**	**0.01**	**0.01**	**0.01**
EX_co2		1.89	1.89	1.89	1.89	1.89	1.89	1.89
EX_erythrose4p		0.01	0.01	0.01	0.01	0.01	0.01	0.01
EX_ethanol		0.68	0.00	0.60	0.40	0.20	0.00	0.00
EX_acetate		**0.07**	0.00	**0.07**	**0.07**	**0.07**	**0.07**	**0.07**
EX_acetoin		**0.06**	0.00	**0.06**	**0.06**	**0.06**	**0.06**	**0.06**
EX_acetaldehyde		0.99	1.86	1.07	1.27	1.47	1.67	1.67

*A, WT reference according to the measurements. B, Theoretically optimal acetaldehyde production in the WT. C, Reduced alcohol dehydrogenase (ADH) activity. High to medium fluxes inside the cells are marked by dark or light red color, respectively. High and medium export or uptake fluxes are marked by dark or light blue color, respectively. D, ADH knockout. E, Enhanced respiration. Bold digits represent fluxes that have fixed boundaries in the respective scenario. Data are given in mol per mol of glucose taken up*.

### Screening of Strains for Elevated Oxygen Consumption and Reduced Alcohol Dehydrogenase Activity

**Figure 2:**
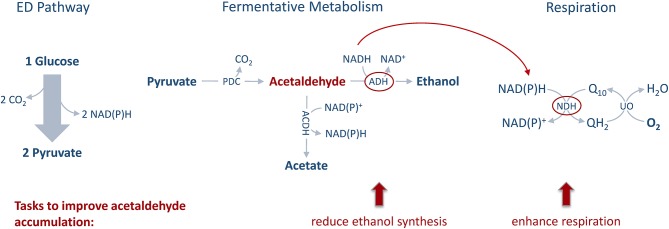
Metabolic engineering approach. The main tasks for improving the acetaldehyde yield of *Z. mobilis* are to reduce ethanol synthesis via alcohol dehydrogenase (ADH) quenching and to elevate respiration by overexpressing NADH dehydrogenase (NDH) and enhancing aeration.

**Figure 3:**
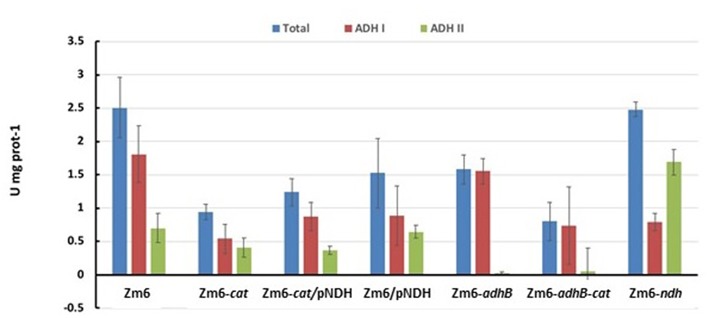
ADH activity after 10 h of aerobic batch cultivation.

**Figure 4:**
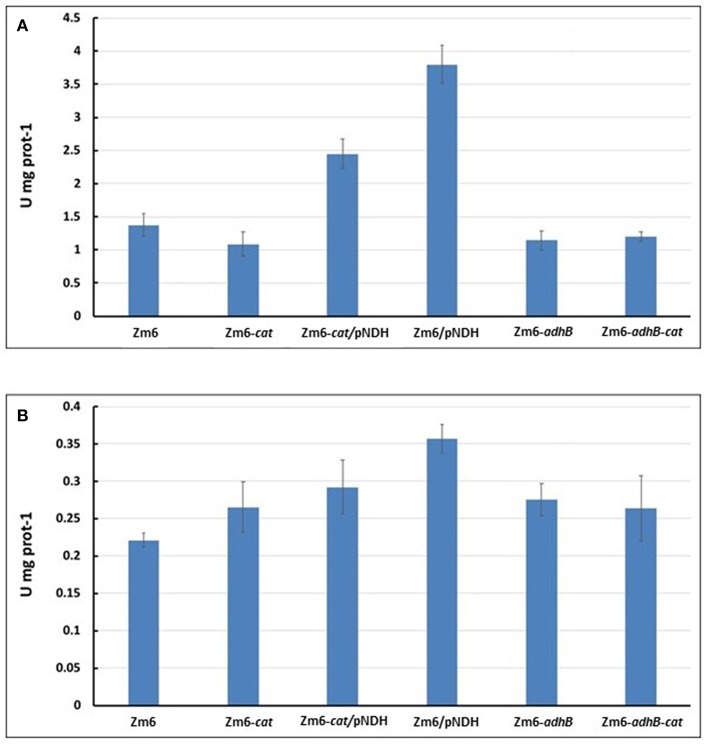
NADH oxidation by Ndh. NADH:CoQ1 oxidoreductase **(A)** and NADH oxidase **(B)** activities in membrane preparations.

**Figure 5:**
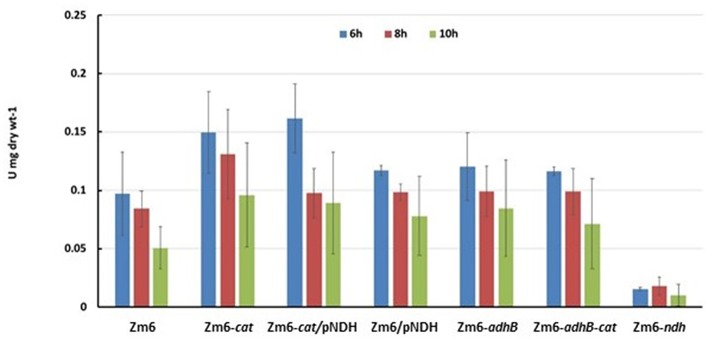
Respiration of late exponential and early stationary state cultures. Oxygen consumption was measured in 1 ml culture samples, directly transferred from the shaken flasks into a Clark electrode chamber without extra substrate addition.

Stoichiometric analysis of *Z. mobilis* central metabolism, based on optimized flux distributions, suggested a roadmap for improving the acetaldehyde yield. The two complementary approaches to take were: (i) raising the respiratory activity and (ii) reducing the alcohol dehydrogenase activity (Figure 2). Accordingly, as the starting point of our study we chose to examine a strain overexpressing the respiratory NADH dehydrogenase (Zm6/pNDH) and a mutant deficient in the iron-containing alcohol dehydrogenase II (Zm6-*adhB*). *Z. mobilis* encodes two alcohol dehydrogenases, the Zn-containing ADH I and the Fe-containing ADH II. Here we did not attempt to construct an ADH double knockout mutant (deficient in both the iron- and zinc-containing isoenzymes), since obtaining and maintaining such a strain might be challenging, due to the reported essentiality of the zinc-containing isoenzyme during the early growth phase (O'Mullan et al., 1995). Zm6/pNDH and Zm6-*adhB* had been constructed previously (see Table 1). The strains were screened for: (i) activities of Fe-containing (ADH II) and Zn-containing (ADH I) alcohol dehydrogenase isoenzymes in cell-free extracts (Figure 3), (ii) NADH oxidation by Ndh, measured as NADH:CoQ1 oxidoreductase (Figure 4A) and NADH oxidase (Figure 4B) activities in membrane preparations, and (iii) respiration of whole cells during aerobic batch growth (Figure 5).

As expected, Zm6-*adhB* had a near-zero activity of the ADH II (Figure 3), while the activity of ADH I was comparable with ADH I activity in Zm6. As a result, Zm6-*adhB* showed a clearly reduced total ADH activity. Compared to the wild-type Zm6, this mutant showed no obvious differences with respect to NADH oxidation (Figure 4) or respiration (Figure 5).

Zm6/pNDH showed an increased NADH oxidation activity (Figure 4) and an increased respiration rate (Figure 5). Notably, the total ADH activity in this strain was reduced and comparable to that of Zm6-*adhB* (Figure 3). This was mainly because of a strongly reduced activity of ADH I. Overexpression of NDH hence reduces ADH activity.

We had previously obtained a catalase mutant (Table 1) and found that in *Z. mobilis* along with the expected increase of hydrogen peroxide sensitivity, catalase deficiency also slightly elevated respiration rate of cells (Strazdina et al., 2012). We therefore included the catalase-negative strain (Zm6-*cat*) in the screen. Performance of Zm6-*cat* was comparable to Zm6/pNDH with respect to the increased respiration rate (Figure 5) and decreased ADH activity (Figure 3).

In addition, we constructed the double knockout strain Zm6-*adhB-cat* and a strain overexpressing NDH against the *cat* background (Zm6-*cat/*pNDH). The WT Zm6 and the NDH-deficient strain (Zm6-*ndh*) were taken for reference. In addition to the near zero activity of ADH II due to the deletion of *adhB*, in Zm6-*adhB*-*cat* also the ADH I activity was low. Thus, this was the strain with the lowest total ADH activity, which was more than three times below that of Zm6 (Figure 3). Notably, Zm6-*ndh* showed an inverted relationship between the isoenzyme activities: a higher ADH II and lower ADH I, than in Zm6.

As expected, strains with the pNDH had the highest activity of NADH:CoQ1 oxidoreductase (Figure 4A), significantly exceeding that of Zm6 (*P* < 0.01 f), and accordingly, the NADH oxidase activity (Figure 4B) also was significantly higher (*P* < 0.01 for Zm6/pNAD). The remaining mutants did not differ from the WT with respect to NADH:CoQ1 oxidoreductase activity, yet showed somewhat elevated NADH oxidase levels (although not meeting the criteria of statistical significance). The NDH-deficient strain had zero activity with NADH (not shown). The observation that the membranes of *cat* and *adhB* mutants had higher oxygen consumption rates with NADH than those of Zm6 (Figure 5), without any increase (rather a slight decrease seen in Zm6-*cat*) of the NADH dehydrogenase activity, implies that NADH dehydrogenase is not the only bottleneck controlling the flux in the electron transport chain of *Z. mobilis*. Finally, oxygen consumption measured in culture samples, which were rapidly transferred from the shaken flasks directly into a Clark electrode chamber without extra substrate addition, showed a somewhat different picture. Again, apart from Zm6-*ndh*, all mutants had slightly higher respiration rates than the wild type, yet strain Zm6/pNDH did not stand out. Instead, the strains Zm6-*cat* and Zm6-*cat/*pNDH showed significantly higher oxygen consumption rates than the WT (*P* < 0.05) during the mid-exponential growth phase (the 6th h in Figure 5). We speculate that such a discrepancy between the whole cell and membrane activities stems from the fact that in whole cells both the respiratory chain activity *per se* and the supply of reducing equivalents to the respiratory chain determine the resulting respiration rate.

### Growth and Synthesis of Acetaldehyde and Other Products

The mutant strains available (Table 1) were systematically characterized with respect to growth kinetics and product synthesis rates for anaerobic growth in closed tubes as well as for aerobic growth (Table 4). To achieve controlled aerobic growth, we chose batch cultivations in bioreactors with a high aeration rate. In these cultivations, pO_2_ typically stays above 60% at all time points assuring availability of oxygen throughout all growth phases.

**Table 4:** Growth rates and byproduct yields of Zm6 and the mutant strains.

**Strain**	**Aerobic growth**			**Anaerobic growth**		
	**Growth rate (h^**−1**^)**	**Y EtOH (mol/mol_**Glc**_)**	**Y AcAld (mol/mol_**Glc**_)**	**Growth rate (h^**−1**^)**	**Y EtOH (mol/mol_**Glc**_)**	**Y AcAld (mol/mol_**Glc**_)**
Zm6	0.38 ± 0.03	0.77 ± 0.06	0.68 ± 0.1	0.42 ± 0.05	1.65 ± 0.16	0.03 ± 0.01
Zm6*-ndh*	0.37 ± 0.03	1.29 ± 0.38	0.04 ± 0.02	0.40 ± 0.04	1.72 ± 0.18	0.01 ± 0.01
Zm6*-cat*	0.34 ± 0.02	0.62 ± 0.18	0.88 ± 0.02	0.39 ±0.03	1.64 ± 0.13	0.04 ± 0.01
Zm6*-adhB*	0.34 ± 0.02	0.52 ± 0.17	1.17 ± 0.11	0.38 ± 0.05	1.61 ± 0.17	0.07 ± 0.01
Zm6*-adhB-cat*	0.31 ± 0.02	0.3 ± 0.1	1.23 ± 0.2	0.38 ± 0.03	1.66 ± 0.04	0.08 ± 0.01
Zm6*/*pNDH	0.33 ± 0.03	0.61 ± 0.16	0.88 ± 0.02	0.41 ± 0.01	1.72 ± 0.06	0.04 ± 0.00
Zm6*-cat/*pNDH	0.28 ± 0.04	0.49 ± 0.06	1.3 ±0.1	0.34 ± 0.01	1.47 ± 0.02	0.09 ± 0.06

Under both conditions, only slight variances in the growth rates of the different strains were observed. Notably, Zm6-*cat*/pNDH, the *cat* mutant strain overexpressing NDH, was growing with the lowest rate under aerobic as well as anaerobic conditions. This mutant is also characterized by the highest acetaldehyde yield under aerobic conditions. Glucose uptake rate was about 40 mmol per gram dry cell weight and per hour, and no significant differences between the strains were observed.

Acetaldehyde production is much more efficient under aerobic growth conditions with only trace amounts being produced anaerobically. Interestingly, the *adhB* and *adhB cat* mutants lacking ADHII activity show the same ethanol yield as Zm6 under anaerobic conditions.

As predicted by the model under aerobic conditions, the ethanol yield was decreased and the acetaldehyde yield was increased in Zm6-*adhB* (Table 4). Increasing the respiration rate by introduction of pNDH also increased the acetaldehyde yield but was less efficient than deletion of *adhB*. In spite of the strong reduction of the total ADH activity in Zm6-*cat* (Figure 3) only a slight increase in acetaldehyde yields was observed for this strain. The combination of the *adhB* and *cat* mutations in Zm6-*adhB*-*cat* resulted in a further improvement of the acetaldehyde yield compared to the *adhB* mutant and in a yield of 1.17 mol acetaldehyde per mole of glucose (Table 3). Introduction of pNDH into Zm6-*cat* resulted in a strong increase in acetaldehyde yield compared to Zm6/pNDH or Zm6-*cat*. In fact, Zm6-*cat*/pNDH showed the best yield of acetaldehyde with 1.3 mole per mole of glucose. The best acetaldehyde producers appeared to be the Zm6-*cat/*pNDH, with the best respiratory capacity and the double mutant Zm6-*adhB*-*cat* with the lowest total ADH activity. Detailed growth data for these strains is shown in Figure 1. Also, the *adhB* strain performed well with respect to acetaldehyde production. While the effect of the *adhB* deletion on ethanol and acetaldehyde production is easily understandable, the effect of the *cat* mutations is less clear.

To analyze the mutant strains in more detail, synthesis of further byproducts of *Z. mobilis* Zm6 was determined. Since no lactate could be detected in our experiments, we focused on measuring acetate and acetoin in addition to ethanol and acetaldehyde.

**Figure 6:**
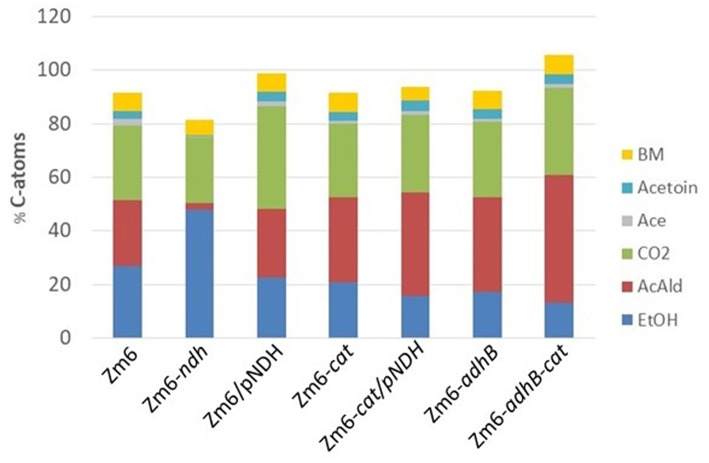
Distribution of carbon atoms from glucose into biomass and by-products. Data were obtained from aerobic bioreactor experiments. The bars represent C atoms from: ethanol (EtOH, blue), acetaldehyde (AcAld, red), carbon dioxide (CO_2_, green), acetate (Ace, gray), acetoin (light blue), and biomass (BM, yellow).

As can be seen from the distribution presented in Figure 6, an almost fixed amount of carbon is converted into biomass. In our hands this is about 5–7%. This is slightly more than the value reported previously (Swings and De Ley, 1977; Rogers et al., 1982; De Graaf et al., 1999). It is interesting to note that evidently the sum of ethanol and acetaldehyde is almost constant in the different strains. However, the ratio of the two products varies a lot. In the *ndh* mutant, almost no acetaldehyde was found. Also, the *ndh* strain did not produce acetate and acetoin in contrast to all other strains. These products hence seem to be related to acetaldehyde production.

### Gene Expression Analysis

To get a better understanding of the processes in the mutant strains, gene expression analysis of Zm6 and the three best acetaldehyde producer strains, the mutants Zm6-*adhB*, Zm6-*adhB*-*cat*, and Zm6-*ca*t/pNDH was performed. As a control, also the *ndh* mutant was analyzed. We selected a set of genes related to central metabolism of *Z. mobilis*. We chose both alcohol dehydrogenases, ADH I and ADH II (encoded by *adhA* and *adhB*), as well as the gene encoding catalase, as we analyzed the respective mutant. NAD dehydrogenase is an important enzyme in order to feed electrons into the respiratory chain of *Z. mobilis* and its expression might change between the mutants. The same holds for lactate dehydrogenase, representing another enzyme able to regenerate NAD and to cytochrome oxidase, being part of electron transport chain. As we were interested in acetaldehyde production, we analyzed additionally the transcription of a gene assumed to encode an acetaldehyde dehydrogenase (Zmob_1390) (Motamedian et al., 2016). Genes of central metabolic pathways, namely *gap, zwf* and *glk* were also analyzed which were not expected to change between the mutant strains. For the sake of completeness, genes representing pyruvate decarboxylase and pyruvate dehydrogenase were also analyzed.

**Figure 7:**
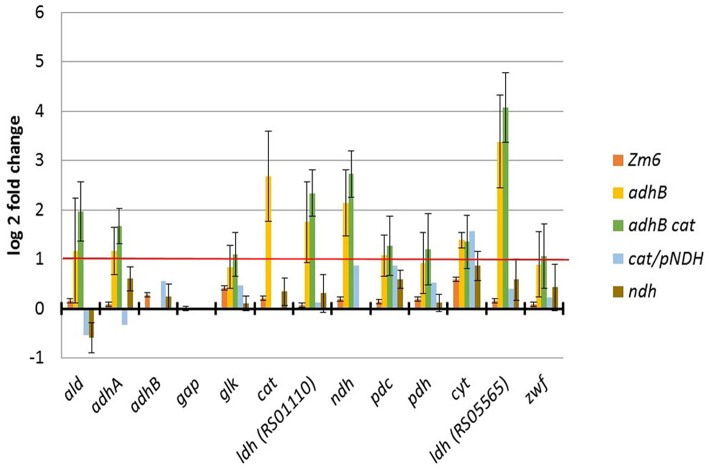
Data from gene expression analysis by real Time RT-PCR. Data were normalized to Zm6 and expression of the housekeeping gene gap. Plotted are data from two or three independent RNA samples (with exception of Zm6-*cat*/pNDH, which was measured only once). Typically, in Real Time RT PCR genes are regarded to be differentially expressed if expression differs by more than a factor of 2. In our case, data seem to be more variable and genes showing a log2-fold change of 1 might still be regarded as being stably expressed.

RNA samples were prepared from the aerobic bioreactor experiments and analyzed via real-time RT-PCR (Figure 7). The data show some notable changes of gene expression in the mutant strains. Compared to Zm6 growing aerobically, *adhA* expression is slightly increased in the strains Zm6-*adhB* and Zm6-*adhB*-*cat*. Most probably, that is because the ADH II (encoded by *adhB*) deficiency is compensated by an increased expression of ADH I. *adhB* expression was not changed in the Zm6-*cat*/pNDH or Zm6-*ndh* mutants, although in Zm6-*cat*/pNDH the ADH II enzymatic activity was lower than in Zm6, while in Zm6-*ndh* it was higher (Figure 3). Notably, in the *adhB* and *adhBcat* mutant strains, *ndh* transcription was strongly increased, although no significant change of the NADH:CoQ1 oxidoreductase activity could be detected in membrane preparations (Figure 4). Likewise, a strongly increased expression was observed for both lactate dehydrogenases tested in the mutants with the *adhB*-negative background. In Zm6-*adhB* also catalase expression was elevated indicating some connection between the regulation of catalase and *adhB* transcription. Taken together, the *adhB*-negative background appears to cause the most of transcriptional difference from the wild type (Figure 7). Inactivation of *adhB* elicited a significantly higher transcription of several dehydrogenases and catalase. As the next step, we chose the two strains with the *adhB*-negative background for a metabolomic study.

### Intracellular Metabolite Levels Under Aerobic Batch Conditions in Zm6 and Three Selected Mutants

Next, we performed profiling of the intracellular metabolite pool in the strains Zm6-*adhB* and Zm6-*adhB-cat*, together with the wild-type Zm6 and the *ndh* mutant as control strains. Metabolomics provide information about responses and adjustments in the intracellular metabolite pools due to genetic changes and can guide strategies for further strain improvements. We measured intracellular concentration of central metabolites including sugar phosphates, nucleosides, and organic acids, using capillary-ion chromatography-tandem mass spectrometry (Kvitvang et al., 2014; Stafsnes et al., 2018).

**Figure 8:**
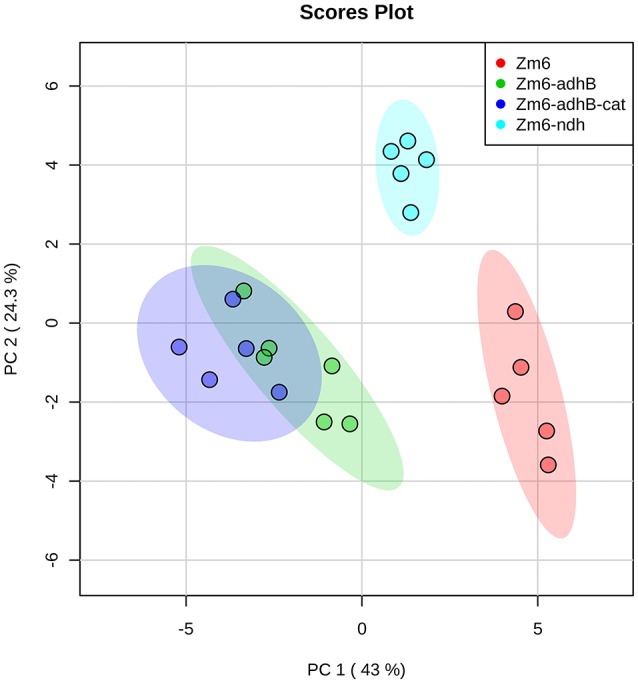
PCA score plot for the analysis of central metabolism in wild-type Zm6, *ndh*-, *adhB*-, and *cat*-*adhB*- mutant strains using a quantitative capIC-MS/MS. The analysis was performed with MetaboAnalyst (Xia et al., 2009). The data were autoscaled prior to PCA.

Preliminary interpretation of the metabolome results using Principle Component Analysis (PCA) revealed some trends between the strains (Figure 8). The *adhB* mutant and the *adhB-cat* mutant cluster together, while Zm6 and the *ndh* mutant exhibited distinctive plots in the analysis. The *ndh* mutant is closer to Zm6 along PC1 (explaining 43% of variance) than the *adhB* mutants, but clearly separated from the WT along PC2 (explaining 25% of variance). This is interesting since Zm6-*ndh* does not produce any acetaldehyde, while both Zm6 and the *adhB* mutants do, although at different levels (Table 4).

The Zm6-*ndh* mutant can maintain the energy charge (adenylate charge, EC) at the same level as Zm6 (0.73 for both), but a drop is observed for the *adhB* mutant and the *adhB cat* double mutant (0.61 and 0.57, respectively; Supplementary Table 1). Interestingly, the drop is not caused by a decrease in ATP but rather a large increase in AMP. The fact that the non-respiring *ndh* mutant has higher EC than the respiring *adhB* mutants and at the same level as Zm6 supports and further underlines the well-known observation about the low energy-coupling efficiency of *Zymomonas* respiratory chain (Rutkis et al., 2016).

**Figure 9:**
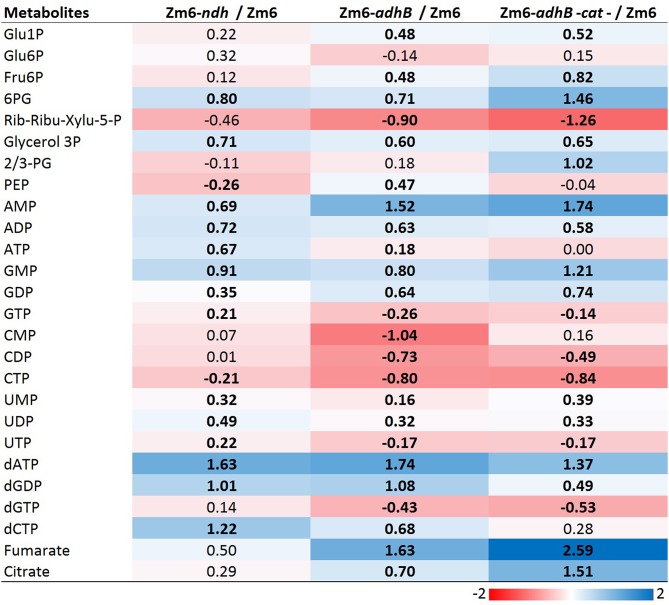
The log2 heatmap visualizes different pattern of ratio to Zm6 between Zm6- *ndh* and Zm6-*adhB* and Zm6-*adhB*-*cat* mutants. Color gradient; scale of log2 value. Black bold font indicates significant changes between individual mutant and WT for that specific metabolite. All the abbreviation of metabolites are as in Supplementary Table 2.

Heat map visualization of the metabolite concentrations in comparison to Zm6 further supports the impression from the PCA score plot of less metabolite pool adjustments in Zm6-*ndh* compared to Zm6-*adhB* background (Figure 9). However, many pools are significantly changed for all three mutants compared to the Zm6 reference strain (*t*-test, *P* < 0.05, red fields). It is possible that *adhB* mutants have adjusted metabolite pools since these strains accumulate acetaldehyde, which possibly will disturb the balance at least between metabolites around the pyruvate and PEP node. Thus, in both strains significantly higher concentrations of fumarate and citrate are seen (Figure 9), implying activity of malic enzyme and PEP carboxykinase, respectively (De Graaf et al., 1999; Pentjuss et al., 2013). Also, acetaldehyde seems to exert a moderate deenergizing effect. Not only the adenylate energy charge, but also the guanylate and uridylate pools in both acetaldehyde-producing strains (and also, citidylate in Zm6-*adhB-cat*) indicate lower levels of the respective nucleotide energy charges (Figure 9). The effect of *ndh* deletion might be more conditional on cultivation conditions and probably closer to the WT strain cultivated under anaerobic conditions with no acetaldehyde production.

Interestingly, PEP, a precursor of pyruvate, was found accumulated in the Zm6-*adhB* (Figure 9). This might be a direct effect of lowered ADH activity, causing lowered flux toward pyruvate by accumulated acetaldehyde, and thus more accumulation of PEP in the *adhB* mutant than in Zm6. However, the same adjustments were not seen in the *adhB-cat* double knock out mutant with no change in PEP but a doubling of 2/3-PG pools (Figure 9). The PEP and 2/3-PG pools are small compared to hexose phosphates, nucleotides, and some TCA metabolites (Supplementary Figure 1). It is interesting that 6-phosphogluconate (6PG) was upregulated in all the measured mutants (Figure 9). 6PG, a metabolite at the branching point between ED pathway and PPP pathway, is not drained away from the ED pathway by oxidative PPP, since pentose phosphates in *Z. mobilis* are generated via the non-oxidative PPP (Jacobson et al., 2019). Therefore, elevated 6PG levels might indicate that ED pathway fluxes are significantly altered in all mutant backgrounds. At the same time, the two strains with the *adhB* background maintain lower pentose phosphate concentration levels. That probably reflects a slow-down of the non-oxidative PPP in these strains.

Although an interpretation of metabolite pool data is challenging and there is no overall direct relationship between metabolite pools and fluxes, our analysis showed that the genetic modifications we introduced caused a significant change of intracellular central metabolism in the mutants.

## Discussion

Due to the special features of *Z. mobilis* central metabolism, namely (i) the high sugar uptakes rates and the rapidly functioning ED pathway along with (ii) the uncoupled aerobic energetics allowing respiration with high rates for regeneration of NAD^+^ and NADP^+^ but low biomass production, this bacterium is conceivably suited as a model organism for acetaldehyde production. In this study, we targeted the aerobic metabolism of *Zymomonas* Zm6 by a rational, model-based metabolic engineering approach to achieve an improved acetaldehyde yield. Investigation of the *Z. mobilis* central metabolism via flux balance analyses using a constraint-based stoichiometric model identified two main tasks for improving the acetaldehyde yield: (1) reduction of ethanol synthesis and (2) enhancement of respiration. The first task was achieved by decreasing ADH activity. The second task was fulfilled by choosing batch cultivation with high aeration and by either overexpressing the respiratory NADH dehydrogenase (pNDH) or by inactivating catalase (*cat*). Catalase was chosen as a target, based on our previously published observation on elevated respiration rate in the *Z. mobilis* catalase knock-out strain (Strazdina et al., 2012). Thus, we initially chose to study aerobic acetaldehyde synthesis in the strains Zm6-*adhB*, Zm6/pNDH, and Zm6-*cat*, taking the wild type Zm6 and the non-respiring Zm6-*ndh* for reference. Besides, aiming to further elevate the mutants' respiration rate, we here constructed and examined a strain overexpressing Ndh in the *cat* background (Zm6-*cat*/pNDH). Unexpectedly, we found that the *cat* mutant background *per se* led to reduced activity in both alcohol dehydrogenase isoenzymes. The total ADH activity in Zm6-*cat* was even lower than that of the *adhB* mutant. This might be explained by a partial inactivation of ADH, especially of ADH I, in the absence of catalase activity (Tamarit et al., 1997). Hence, it turned out that catalase inactivation played a dual role in our engineering strategy: it both, improved the respiration rate and served to decrease ADH activity. Based on this finding and striving to further decrease the total ADH activity, we constructed also the double mutant Zm6-*adhB-cat*, and included it in the screen.

The total alcohol dehydrogenase activity in *Z. mobilis* represents the sum of activities of two biochemically well-characterized ADH isoenzymes, ADH I and II (Conway et al., 1987b; Keshav et al., 1990). In the study at hand, we used a knock-out mutant of ADH II encoded by *adhB*. The *adhB* mutant was deficient for the Fe-containing oxygen sensitive ADH II and apparently exhibited lower total ADH activity. The remaining ADH activity in this strain was comparable to the ADH I activity measured in Zm6. Acetaldehyde synthesis in an ADH II-deficient *Z. mobilis* strain has been studied previously (Wecker and Zall, 1987). Close to 40% of the maximum theoretical acetaldehyde yield was reached from glucose. However, Wecker and Zall obtained their ADH II-deficient strain by applying selection with allyl alcohol, and did not provide its genetic characteristics. Generally, this method allows for the selection of bacteria with impaired ability to oxidize alcohol. That implies selective pressure for ADH-deficiency, but also for other mutations, like some respiratory defects (Kalnenieks, unpublished observation). Our present work is the first attempt to study acetaldehyde production with an *adhB* mutant background, constructed by homologous recombination (Kalnenieks et al., 2006).

As expected, all introduced mutations influence acetaldehyde production. The *ndh* mutant showed almost no acetaldehyde production while it was strongly increased in Zm6-*adhB*, Zm6-*adhB-cat* and Zm6-*cat*/pNDH mutants. Our experimental data hence confirmed the model simulations, showing that a decrease in ADH activity (Zm6-*adhB*, Zm6-*cat*, Zm6-*adhB-cat*) or an increase in respiration (NDH overexpression, Zm6-*cat*) enhances acetaldehyde production. We were able to demonstrate that the energetically inefficient electron transport chain of *Z. mobilis* could be used for redox balancing purposes. An enhanced flux through the electron transport chain withdrew NADH from the alcohol dehydrogenase reaction, resulting in diminished ethanol production in favor of acetaldehyde. As a result, we achieved acetaldehyde yield values of 66% of the theoretical maximum for Zm6-*adhB*-*cat* and 70% for Zm6-*cat/*pNDH (with 1.86 mol/mol_Glc_ regarded as 100%), which is the highest acetaldehyde yield reported for microorganisms so far.

Notably the introduced mutations affected gene expression and concentrations of central metabolites to a various degree. In general, inactivation of *adhB* elicited more profound effects than the *cat* knock-out or variation of Ndh background. In both strains with the *adhB* negative background, we observed (i) an increased transcription of several dehydrogenase genes and the catalase gene, (ii) a substantial change of the E-D, PPP and TCA intermediate concentrations, and (iii) a decrease of cellular energy charge. Apparently, those are the consequences of a direct interference with the “catabolic highway” of this bacterium, an intrinsic part of which is the activity of ADH II. For further improvement of acetaldehyde yield, the stoichiometric model suggests inactivation of both ADH isoenzymes. However, that might be a challenging task, putting even more stress upon the rest of central metabolism and possibly affecting viability.

Apart from *Z. mobilis*, there have been successful attempts to engineer *Lactococcus lactis* (Bongers et al., 2005) and *Escherichia coli* (Balagurunathan et al., 2018) for acetaldehyde production from sugary substrates. Aiming to improve acetaldehyde yield, pyruvate decarboxylase of *Z. mobilis* was overexpressed in both bacteria. Since neither of these bacteria possess a low energy-coupling respiratory chain comparable to that of *Z. mobilis*, also the soluble NADH oxidase (Nox) from *L. lactis* was overexpressed, to ensure rapid aerobic oxidation of NADH. In resting cells of engineered *L. lactis* conversion of almost 50% of glucose to acetaldehyde was demonstrated, reaching 0.95 moles of acetaldehyde per mole of glucose (Bongers et al., 2005), although substantial amounts of lactate and other catabolic byproducts were accumulated as well. In the engineered *E. coli* improvement of acetaldehyde production required knocking out of several competing metabolic pathways. As a result, 37% of the theoretical maximum yield was reached (Balagurunathan et al., 2018). Acetoin was produced as a major byproduct by the carboligase activity of the introduced *Z. mobilis* pyruvate decarboxylase and ethanol was another byproduct.

In contrast to other acetaldehyde producers, in *Z. mobilis* we encountered fewer problems with aerobic byproduct accumulation. Acetate production started late during batch fermentation, after the maximum of acetaldehyde production had been reached (see Figure 1), and hence, it almost did not interfere with acetaldehyde production. Unexpectedly, acetoin was a minor byproduct (Figure 6), in sharp contrast to *E. coli*, expressing the same *Z. mobilis* pyruvate decarboxylase (see above). Based on the product measurements, we conclude that for *Z. mobilis* the total amount of carbon, incorporated into the ethanol/acetaldehyde pool is nearly constant (Figure 6), but the ratio of the two metabolites shifts depending on the capacity of the ADH fluxes and the flux through the electron transport chain. There are further options to raise the respiratory rate and further decrease the alcohol dehydrogenase activity, as well as to inactivate the enzyme(s), producing acetate during the late phase of batch culture. Yet, even more important for further improvement of acetaldehyde yield and productivity would be to find ways to efficiently remove this volatile inhibitory compound from the fermentation medium. Metabolic engineering of acetaldehyde producers very much follows the same logic as that of engineering microbial strains for synthesis of longer chain aldehydes. Engineered *E. coli*, producing *n*-butyraldehyde (Ku et al., 2017) or isobutyraldehyde (Rodriguez and Atsumi, 2012) may serve as relevant examples. As with acetaldehyde, in both cases production could be improved by inactivating enzymes, converting aldehydes to the respective alcohols, and by oxidizing the excess reducing equivalents. Furthermore, production was increased by efficient removal of the inhibitory products from fermentation medium, either by a gas-stripping system (Rodriguez and Atsumi, 2012), or by *in situ* liquid-liquid extraction (Ku et al., 2017).

## Data Availability Statement

All datasets generated for this study are included in the article/Supplementary Material.

## Author Contributions

EB, IS, and RR were involved in strain construction, cultivation, and enzyme assays. KB and SS performed strain characterization and gene expression analysis. KF and PB performed metabolite analysis. JR, OS, AP, and RR were involved in model set up and analysis. UK, JR, KF, PB, RR, RP, KP, and KB participated in the design of the experiments and in the interpretation of results and in writing of the manuscript.

### Conflict of Interest

The authors declare that the research was conducted in the absence of any commercial or financial relationships that could be construed as a potential conflict of interest.
