# Supplementary material for: Improvement of Acetaldehyde Production in Zymomonas mobilis by Engineering of Its Aerobic Metabolism
Source: Front Microbiol. 2019 Nov 14;10:2533. doi: 10.3389/fmicb.2019.02533 (PMC6868117; doi:10.3389/fmicb.2019.02533)
Supplement: Supplementary file 3 [file Image_1.pdf]

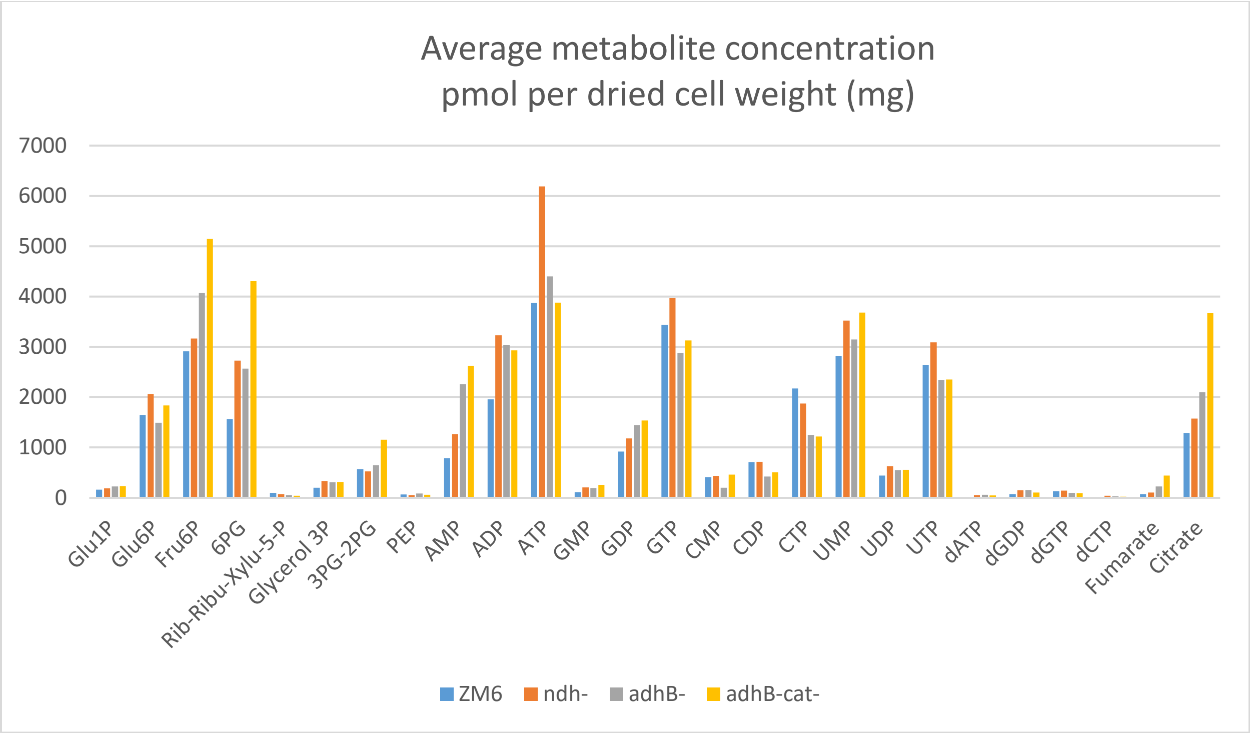

Supplementary Figure 1

Comparison of measured metabolites between wild-type ZM6, *ndh*-, *adhB*-, and *cat*-*adh*B- mutant strains. Unit for Y axis; pmol per dried cell weight (mg).
